# Development and initial validation of a traditional Chinese medicine symptom-specific outcome measure: a *Zheng*-related atopic dermatitis symptom questionnaire (ZRADSQ)

**DOI:** 10.1186/1477-7525-11-212

**Published:** 2013-12-21

**Authors:** Darong Wu, Chujun Huang, Xiumei Mo, Junfeng Liu, Jianxiong Cai, Chi Liu, Haili Zhu, Hongyi Li, Dacan Chen

**Affiliations:** 1 The 2nd Affiliated Hospital of Guangzhou University of Chinese Medicine, Guangzhou, China; 2 Department of Clinical Epidemiology & Biostatistics, McMaster University, Hamilton, Canada; 3 Beijing Aerospace General Hospital, Beijing, China; 4 The 2nd Affiliated Hospital of Guangzhou University of Chinese Medicine, 111 Dade Road, PO Box 510120, Guangzhou, China

**Keywords:** Atopic dermatitis, Development, Questionnaire, Traditional Chinese medicine, Validation, Symptom

## Abstract

**Background:**

*Zheng* represents pattern differentiation in Traditional Chinese Medicine (TCM), as the basic unit and a key concept in TCM therapeutic theory, is based on the physiology and pathology of TCM. None of the outcome measurements of atopic dermatitis (AD) are *Zheng*-specific. The effectiveness of TCM is likely to be underestimated without a *Zheng*-related symptom-specific instrument. The aim of this study was to develop an instrument for measuring the *Zheng*-related symptom-specific status of patients with AD.

**Methods:**

We followed standard methodology to develop the instrument, including item generation and selection, item reduction and presentation, and pretesting, and recruited 188 patients with AD involved in a six-center randomized-controlled trial (ChiCTR-TRC-08000156) to validate the questionnaire. We conducted construct validity, reliability, and responsiveness analysis. The standardized effect size (SES) and standardized response mean (SRM) were used to calculate the responsiveness of additional items and the total score for the rating items.

**Results:**

ZRADSQ has 15 items, with 12 rating items and 3 additional items. The 12 rating items fall within three domains: AD symptoms (n = 6 items); Heat (n = 4 items) and Mood (n = 2 items). Confirmatory factor analysis provided good support for a three-factor model (d.f. = 51, *x*^2^=97.11, RMSEA = 0.07, CFI = 0.96), and the Pearson’s correlation coefficient between ZRADSQ and Severity Scoring of Atopic Dermatitis (SCORAD) was 0.40 (*P* < 0.001). The reliability was also good, with a Cronbach’s alpha value for ZRADSQ of 0.84, a split-half coefficient of 0.75, and a test-retest reliability coefficient of 0.98. The standardized effect size and standardized response mean were close to or larger than 1, which indicated moderate to good responsiveness.

**Conclusions:**

The ZRADSQ demonstrates promising reliability, validity, and responsiveness. It can be used to determine whether *Zheng*-specific or symptom-specific treatments relieve the symptom that is most bothersome the patient.

## Background

Atopic dermatitis (AD) is a commonly occurring chronic inflammatory skin disease that affects an estimated 10-20% of children and 1-3% of adults [1]. It is reported that in the United States the direct cost of AD treatment may exceed $3 billion per year [2]. Traditional Chinese Medicine (TCM), as one of the therapeutic concepts of Complementary or Alternative Medicine (CAM), is becoming increasingly popular for the treatment of inflammatory skin diseases, primarily atopic dermatitis (AD) [3], such as herbal medicine, massage, and acupuncture. A survey found that among patients with AD, 42.5% had use alternative therapies [4], including herbal remedies, homeopathy.

Valid and reliable outcome measurements are a prerequisite for evidence-based practice [5]. However, no laboratory tests have been conducted to adequately assess disease severity in AD. Consequently, several outcome measures for AD have been published. Only the Severity Scoring of Atopic Dermatitis (SCORAD), Eczema Area and Severity Index (EASI), and Patient-oriented Eczema Measure (POEM) measures currently perform adequately [6], but none of these is *Zheng* specific or *Zheng*-related symptom specific.

*Zheng*, which is also called syndrome or pattern, is a basic concept in TCM. The effectiveness of TCM largely depends on whether the treatment is coordinated with *Zheng *[7]*.* Yan et al. evaluated 19 clinical trials and found that ginseng had a positive effect on participants with Qi deficiency *Zheng* (pattern), but a negative effect on participants without Qi deficiency *Zheng* (pattern) [8]. Pattern diagnosis as *Zheng* differentiates clinical symptom-complexes, and represents specific conditions that can be adjusted or reversed by corresponding TCM therapeutic techniques such as herbal medicine and acupuncture. Patients with the same illness may have varying symptom-complexes because of individual differences in health, and are managed by TCM *Zheng*-specific or *Zheng*-related symptom-specific therapies [9]. However, without a *Zheng*-specific or *Zheng*-related symptom-specific instrument, the effectiveness of TCM *Zheng*-specific therapies is likely to be underestimated.

The aim of this project was thus to develop an instrument for measuring *Zheng*-related symptom-specific status in patients with AD.

## Methods

**Figure 1:**
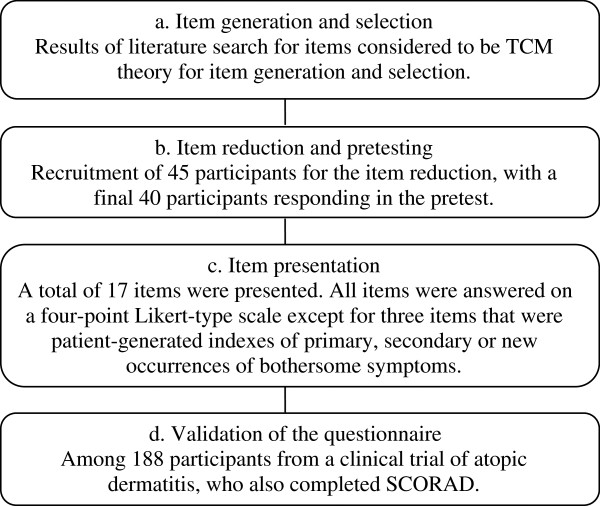
Flow chart of the study phases.

We followed standard methodology for instrument development, which is divided into the phases of item generation and selection, item reduction and presentation, pretesting, and questionnaire validation (see Figure 1). The item generation phase was based on information gathered from ancient and modern literature.

### Item generation and selection

We began our work by searching the literature, both ancient and modern, for descriptions of AD *Zheng-*related symptoms that could inform our work. We searched two databases for the modern literature – the Chinese Bio-medicine Database (CBMdisc, 2003–2008) and the China Scientific Journals Full-text Database (CSJD, 2003–2008) – using “dermatitis/atopic” as the subject terms with all of the subheadings. The keywords used for the ancient literature search included *TaiLianChuang* (胎敛疮), *NaiXuan* (奶癣), *RuXuan* (乳癣), *XueFengChuang* (血风疮), *SiWanFeng* (四弯风), *JinYinChuang* (浸淫疮), *TaiXuan* (胎癣), *LianMeiChuang* (恋眉疮) and *LianMeiChuang* (炼眉疮), which were obtained from twelve textbooks or books, such as *Dermatovenerology of Integrated Traditional and Western Medicine* and *External Medicine of Traditional Chinese Medicine*. Publications up to 1919 were searched in the Encyclopaedia of Traditional Chinese Medicine (CD-ROM Updated Version, 2006).

**Figure 2:**
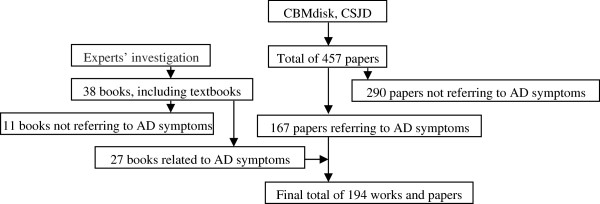
Modern literature search process.

**Figure 3:**
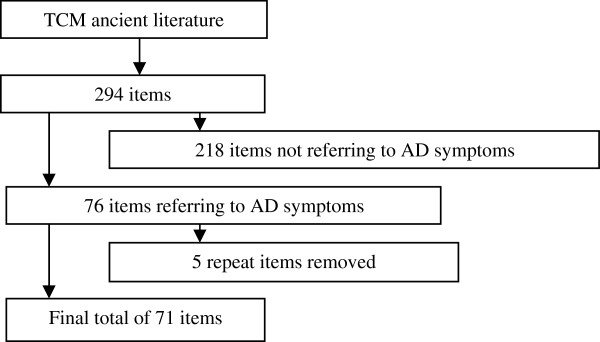
Ancient literature search process.

The literatures on *Zheng*-related symptoms of AD that formed the basis for the item generation contained 194 articles from modern studies, textbooks, and works, and 294 paragraphs from classical literatures, including works such as “Discussion of Various Causes of Disease Periods of Five Days (Sui dynasty)”, “Golden Mirror of Medicine (Qing dynasty)”, and “Prescriptions for Universal Relief (Ming dynasty)” (see Figures 2 and 3). Items from these articles or paragraphs largely overlapped, and so we grouped them into similar themes. Only items with a frequency of more than 2% were selected.

### Item reduction and pretesting

Experts in dermatology and clinical epidemiology took part in the process of item reduction and presenting. Total of 18 items were presented, including 15 rating items, and 3 additional items that were patient-generated indexes of primary, secondary, and new occurrences of bothersome symptoms. Forty patients with AD from the Out-patient department, 2^nd^ Affiliated Hospital of Guangzhou University of Chinese Medicine, Guangzhou, responded for the item reduction and initial pretesting phases. Table 1 shows the demographic characteristics of these participants. All of the participants met the Raika and Hanifin AD diagnostic criteria [10] and were aged between 7 and 25 years old. The participants used a four-point Likert-type scale to rate their AD symptoms for the 15 selected rating items, where 0 = none, 1 = mild, 2 = moderate, and 3 = severe. They were also asked to report the primary bothersome symptom in the past week, and any secondary bothersome symptoms. At the next visit, the participants were asked to report whether they had experienced any newly occurring bothersome symptoms.

**Table 1:** General information on the participants in the pretesting phase (N = 40)

		**n(%)**
Age(year)	7-12	20(50%)
	13-16	8(20%)
	17-25	12(30%)
Sex	Male	19(47.5%)
	Female	21(52.5%)
Atopic disease history	Yes	29(72.5%)
Parents with atopic disease history	Yes	17(42.5%)

We conducted descriptive analysis and tested the item discrimination coefficients for all of the rating items by dividing the participants into two groups – one group with larger item scores of more than 27%, and another group with lower item scores of less than 27% [11-13] – and used an independent samples t-test to compare the two groups. If the result was *p*< 0.05, then the item was deemed to be good at discrimination; if not, then the item was deemed to be less discriminative. The item-total correlations for the rating items were also calculated (the additional items were not included). Items with a low discrimination coefficient or with a low item-total correlation were eliminated.

### Item presentation

Base on the results of the item discrimination coefficients and the item-total correlations, one item was deleted. All 17 items (14 rating items and 3 additional items) emerging from the item reduction were answered on a four-point Likert-type scale, except for the three additional items about bothersome symptoms. The participants were asked about their symptoms in the past week, and showed ease of understanding of the items. There were no ceiling or floor effects for the 14 rating items. The instrument required less than 9 minutes (an average of 8.25 minutes) to complete.

### Validation of the questionnaire

We recruited another sample of patients with AD involved in a six-center randomized-controlled trial (ChiCTR-TRC-08000156). All of the participants met the Hanifin and Rajika AD diagnostic criteria [10], had moderate to severe AD that had lasted more than one year, and were aged between 5 and 25 years old. In this trial, there were 275 eligibility, 250 patients were included, while 30 patients did not receive intervention and 14 patients were dropped out. Among the 206 subjects who completed the whole period of intervention, 188 patients were per protocol. They completed the 17-item initial version of the *Zheng*-Related Atopic Dermatitis Symptom Questionnaire (ZRADSQ) and SCORAD [14]. In the nine-month duration of the trial, the participants were required to complete the ZRADSQ and SCORAD at the same time on baseline, week 4, week 8, and week 12. The ZRADSQ was self-reported by the patients or reported by their parents if they were less than seven years old.

### Statistical analysis of validity and reliability

The face validity was judged by the investigators [15]. For the rating items, confirmatory factor analysis (CFA) was used to test a model with three domains as initially designed and to eliminate items or revise the structure of the questionnaire based on a root mean square error of approximation (RMSEA) of less than or equal to 0.08 and a comparative fit index (CFI) of equal to or greater than 0.95 [16]. Using the revised questionnaire, we evaluated the convergent construct validity by calculating the Pearson’s correlation coefficients between the ZRADSQ and SCORAD. We calculated the Cronbach’s alpha values to investigate the internal consistency of the ZRADSQ domains [15], using a split-half coefficient (Unequal-length-Spearman-Brown coefficient) to evaluate the split half consistency. We considered correlations of more than 0.35 to 0.50 to be moderate, more than 0.50 to be strong, less than 0.35 to be weak, and less than 0.20 to be very weak. We used the Pearson’s correlation coefficient to take repeated measurements in a sample of 30 pretest patients (part of the sample of 40 individuals) who completed the questionnaire twice within 48 hours to evaluate the test-retest reliability of the ZRADSQ. And we used a paired t-test to calculate the discriminant validity. For the additional items, we used the standardized effect size (SES) and standardized response mean (SRM) to calculate the responsiveness characteristic of two of the additional items and the total score of the rating items. The SES and SRM were the two indexes used to judge responsiveness, with a value close to or larger than 1 indicating moderate to good responsiveness. We divided the participants into two subgroups due to their age, i.e. one subgroup with subjects equal or less than 7 years old, another larger than 7 years old. We also calculated the standards association validity coefficient, Cronbach’s alpha coefficient, split-half coefficient, paired t-test, SES, SRM respectively in these two subgroups. We used Lisrel 8.8 (Scientific Software International, Inc, Chicago) for the CFA and SPSS for Windows 17.0 for the other statistical analyses (SPSS, Inc, Chicago).

### Ethics

The validation study design was approved by the Ethics Committee of the 2^nd^ Affiliated Hospital of Guangzhou University of Chinese Medicine (i.e. Guangdong Provincial Hospital of Chinese Medicine), Guangzhou, Guangdong Province, China (Approved No.2008GL-09). The recruited participants signed an informed consent form. If the patient was younger than 18 years old, then the form was signed by the patient’s parents.

## Results

### Development

Forty participants, from the out-patient department, the 2^nd^ Affiliated Hospital of Guangzhou University of Chinese Medicine, completed the pretest questionnaire of 18 items. Their descriptive characteristics are shown in Table 1. The number of items was reduced to 17 through statistical analysis in the item reduction phase (see Table 2). In the discrimination coefficient analysis, the *p* value was less than 0.05 for all of the items.

**Table 2:** Results of the item-total correlation analysis in the item reduction phase (N = 40)

**Item**	**Scale mean if item deleted**	**Scale variance if item deleted**	**Corrected item-total correlation**	**Cronbach’s alpha if item deleted**
Q1 Itching	15.48	77.18	0.48	0.88
Q2 Itching accompanied by pain	16.60	74.35	0.60	0.87
Q3 Pain	17.03	74.74	0.54	0.88
Q4 Itching aggravated at night	15.68	73.82	0.51	0.88
Q5 Dry skin	15.45	75.90	0.57	0.87
Q6 Night sweats	17.10	80.66	0.20*	0.89
Q7 Burning skin	16.75	72.30	0.55	0.88
Q8 Fatigue	16.80	73.20	0.56	0.87
Q9 Fidgeting	16.63	69.68	0.72	0.87
Q10 Irascibility	16.75	69.73	0.67	0.87
Q11 Insomnia	17.00	73.13	0.58	0.87
Q12 Mouth dryness	16.88	5.80	0.58	0.87
Q13 Thirst	16.95	74.77	0.62	0.87
Q14 Dark urine	16.98	74.38	0.58	0.87
Q15 Constipation	17.15	78.18	0.39	0.88

*Note: Q6 night sweats was deleted (Corrected Item-Total Correlation = 0.195).

### Validation

**Figure 4:**
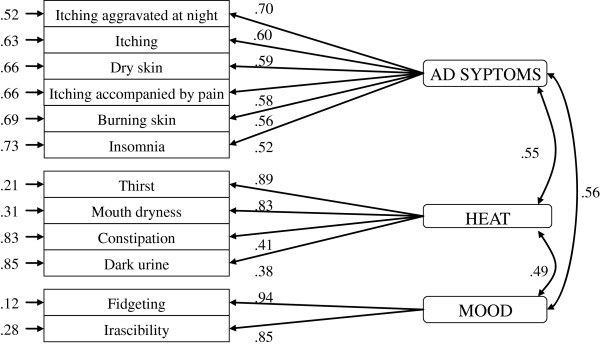
Parameter estimate values of the model.

For the 188 per protocol subjects of the trial, forty-eight percent of the participants were male. The mean age of the recruited participants was 13.57 years. Table 3 shows the general information on the participants. Among the 17 items (14 rating items and 3 additional items), the 14 rating items were introduced into the initial CFA model. Two items, Q3 (pain) and Q8 (fatigue), were eliminated due to a relatively decreasing amount of RMSEA if they were removed from the model. The final model with twelve items and three domains had 51 degrees of freedom and a chi-square value of 97.11. The RMSEA of this model was 0.07, which is less than the cutoff of 0.08, and the CFI was 0.96. All of these results indicate that the model is valid (see Figure 4). The final version of instrument, ZRADSQ, was with 12 rating items and 3 additional items.

**Table 3:** General information on the participants in the validation phase (N = 188)

	**Group**	**Number**	**%**
Age (year)	>18	50	26.6
	14-18	30	16.0
	<14	108	57.4
Sex	Male	91	48.4
	Female	97	51.6
Duration of disease (year)	>10	67	35.6
	5-10	75	40.0
	<5	46	24.4
Atopic disease history	Yes	93	49.5
Parents with atopic disease history	Yes	29	15.4
Marriage status	Unknown	1	0.5
	Unmarried	183	97.4
	Married	4	2.1
Sites*	Guangzhou (1st)	70	37.2
	Chengdu	31	16.5
	Nanjing	29	15.4
	Guangzhou (2nd)	13	6.9
	Haikou	31	16.5
	Luzhou	14	7.5

*Note: Two sites were located in Guangzhou.

Tables 4, 5 and 6 shows that the Pearson’s correlations between the ZRADSQ and SCORAD in total and different age groups were ranged from 0.39-0.47 (*P* < 0.02).

**Table 4:** Standards association validity coefficient results in the validation phase (N = 188, all ages)

	**Kolmogorov-smirnov**			**Pearson correlation coefficient**	
	**Statistic**	**df**	***P***	**Statistic**	***P***
ZRADSQ	0.06	188	0.06	0.40	<0.001
SCORAD	0.08	188	0.01		

**Table 5:** Standards association validity coefficient results in the validation phase (N = 25, age ≤ 7)

	**Kolmogorov-smirnov**			**Pearson correlation coefficient**	
	**Statistic**	**df**	***P***	**Statistic**	***P***
ZRADSQ	0.17	25	0.08	0.47	0.02
SCORAD	0.13	25	0.20		

**Table 6:** Standards association validity coefficient results in the validation phase (N = 163, age>7)

	**Kolmogorov-smirnov**			**Pearson correlation coefficient**	
	**Statistic**	**df**	***P***	**Statistic**	***P***
ZRADSQ	0.06	163	0.20	0.39	<0.001
SCORAD	0.08	163	0.01		

### Reliability

The Pearson correlation coefficient between the first and repeat measurements was 0.98 (see Table 7). The total Cronbach’s alpha coefficient was 0.84, with a range of 0.74-0.89 for the individual domains (see Table 8), and similar results were obtained in the two subgroups, i.e. one with participants equal or less than seven years old, another with those larger than seven years old (see Table 9 and Table 10 respectively). The results for the split-half coefficient of total questionnaire and the two age groups were shown in Tables 11, 12 and 13.

**Table 7:** Test-retest reliability results in the validation phase (N = 30)

	**Kolmogorov-smirnov**			**Shapiro-wilk**			**Pearson’s correlation coefficient**	
	**Statistic**	**df**	***P***	**Statistic**	**df**	***P***		***P***
First measurement	0.14	30	0.18	0.96	30	0.37	0.98	<0.001
Repeat measurement	0.13	30	0.20	0.97	30	0.50		

**Table 8:** Cronbach’s alpha coefficient in the validation phase (N = 188, all ages)

	**Cronbach’s alpha**	**No. of items**
ZRADSQ	0.84	12
AD symptoms	0.75	6
Heat	0.74	4
Mood	0.89	2

**Table 9:** Cronbach’s alpha coefficient in the validation phase (N = 25, age ≤ 7)

	**Cronbach’s alpha**	**No. of items**
ZRADSQ	0.89	12
AD symptoms	0.85	6
Heat	0.73	4
Mood	0.94	2

**Table 10:** Cronbach’s alpha coefficient in the validation phase (N = 163, age>7)

	**Cronbach’s alpha**	**No. of items**
ZRADSQ	0.83	12
AD symptoms	0.73	6
Heat	0.74	4
Mood	0.88	2

**Table 11:** Split-half coefficient in the validation phase (N = 188, all ages)

	**No. of items**	**Cronbach's alpha**	**Spearman-brown coefficient**	
			**Equal length**	**Unequal length**
Part 1	6	0.75	0.75	0.75
Part 2	6	0.77		

Part 1: Itching aggravated at night, itching, dry skin, itching accompanied by pain, burning skin, insomnia.

Part 2: Thirst, mouth dryness, constipation, dark urine, fidgeting, irascibility.

**Table 12:** Split-half coefficient in the validation phase (N = 25, age ≤ 7)

	**No. of items**	**Cronbach's alpha**	**Spearman-brown coefficient**	
			**Equal length**	**Unequal length**
Part 1	6	0.85	0.91	0.91
Part 2	6	0.74		

Part 1: Itching aggravated at night, itching, dry skin, itching accompanied by pain, burning skin, insomnia.

Part 2: Thirst, mouth dryness, constipation, dark urine, fidgeting, irascibility.

**Table 13:** Split-half coefficient in the validation phase (N = 163, age>7)

	**No. of items**	**Cronbach's alpha**	**Spearman-brown coefficient**	
			**Equal length**	**Unequal length**
Part 1	6	0.73	0.72	0.72
Part 2	6	0.78		

Part 1: Itching aggravated at night, itching, dry skin, itching accompanied by pain, burning skin, insomnia.

Part 2: Thirst, mouth dryness, constipation, dark urine, fidgeting, irascibility.

### Discriminant validity and responsiveness

For both primary and secondary bothersome symptoms, the ZRADSQ and SCORAD showed significant differences between the baseline and Week 12 scores, with SES values ranging from 0.94 to 1.38 and SRM values ranging from 1.00 to 1.35. However, the SES and SRM values were smaller in those who were equal or less than 7 years old. (see Tables 14, 15 and 16).

**Table 14:** Results of the paired t-test, SES, SRM in the validation phase (N = 188, all ages)

	**Baseline**	**Week 12**	**Difference between before and after treatment**				**SES**	**SRM**
	***x***^***- ***^ ***± SD***	***x***^***- ***^ ***± SD***	***x***^***- ***^ ***± SD***	***P***	**T**	**95% CI**		
Primary bothersome symptom	6.96 ± 2.22	3.89 ± 2.50	3.06 ± 2.86	<0.001	14.68	2.65 - 3.48	1.38	1.07
Secondary bothersome symptom	6.37 ± 2.34	3.50 ± 2.45	2.87 ± 2.88	<0.001	13.67	2.46 - 3.29	1.23	1.00
ZRADSQ	15.14 ± 6.74	8.78 ± 5.62	6.35 ± 6.29	<0.001	13.84	5.45 - 7.26	0.94	1.01
SCORAD	47.61 ± 15.57	26.85 ± 15.56	20.76 ± 15.34	<0.001	18.56	18.55 - 22.97	1.33	1.35

**Table 15:** Results of the paired t-test, SES, SRM in the validation phase (N = 25, age ≤ 7)

	**Baseline**	**Week 12**	**Difference between before and after treatment**				**SES**	**SRM**
	***x***^***- ***^ ***± SD***	***x***^***- ***^ ***± SD***	***x***^***- ***^ ***± SD***	***P***	**T**	**95% CI**		
Primary bothersome symptom	6.68 ± 1.88	4.04 ± 2.43	2.64 ± 2.03	<0.001	6.49	1.80-3.48	1.40	1.30
Secondary bothersome symptom	6.35 ± 2.15	3.99 ± 2.43	2.36 ± 2.27	<0.001	5.21	1.42-3.30	1.10	1.04
ZRADSQ	15.28 ± 7.86	9.72 ± 5.36	5.56 ± 7.35	<0.001	3.78	2.52-8.60	0.70	0.76
SCORAD	42.87 ± 14.38	26.04 ± 14.50	16.83 ± 15.85	<0.001	5.31	10.29-23.38	1.17	1.06

**Table 16:** Results of the paired t-test, SES, SRM in the validation phase (N = 163, age>7)

	**Baseline**	**Week 12**	**Difference between before and after treatment**				**SES**	**SRM**
	***x***^***- ***^ ***± SD***	***x***^***- ***^ ***± SD***	***x***^***- ***^ ***± SD***	***P***	**T**	**95% CI**		
Primary bothersome symptom	7.00 ± 2.27	3.87 ± 2.52	3.13 ± 2.97	<0.001	13.45	2.67-3.59	1.38	1.05
Secondary bothersome symptom	6.37 ± 2.38	3.42 ± 2.45	2.95 ± 2.96	<0.001	12.72	2.49-3.41	1.24	1.00
ZRADSQ	15.12 ± 6.58	8.63 ± 5.66	6.48 ± 6.14	<0.001	13.48	5.53-7.43	0.98	1.06
SCORAD	48.34 ± 15.66	26.97 ± 15.76	21.36 ± 15.22	<0.001	17.93	19.01-23.72	1.36	1.40

For the primary and secondary bothersome symptoms, patients were more likely to report itching (34.73%), dry skin (21.41%), itching aggravated at night (20.10%), itching accompanied by pain (9.40%), and fidgeting (3.92%) than the other items. Only a few patients present other symptoms, such as red skin, skin are not beautiful to look at, skin erosion and skin exudation.

### Scoring system

The scoring system of this instrument contains two parts. One is the independent VAS for the primary, secondary, and new occurrences of bothersome symptoms for the 3 additional items, range from 0.0 to 10.0, the higher the worse; the other one is a four-point Likert-type scale for the 12 rating items, values with 0, 1, 2, 3 representing None, Mild, Moderate, Severe symptoms respectively, and the sum of them is total score, higher score indicates worse status.

## Discussion

We developed an instrument that evaluates *Zheng*-related symptoms for use among patients with atopic dermatitis (AD) by applying standard methodology and following an established framework. The 15-item ZRADSQ (see Additional file 1) shows good face validity, adequate internal consistency reliability, and good responsiveness in detecting change. The construct validity evaluation indicated correlations with SCORAD were coordinated with the estimated magnitude and direction.

Some studies on AD measurements using the SCORAD as the criterion [17], for example, Hon et al. validated the Chinese version of the Nottingham Eczema Severity Score have good agreement and correlation with SCORAD [18]. Although SCORAD is a good instrument for AD, it was also used to be the measurement of efficacy and patient’s condition in the trials of Complementary or Alternative Medicine (CAM) domain, such as Traditional Korean Medicine, TCM and homoeopathic treatment for AD[19-21], however, a huge difference, especially if underestimated in the mild group, would occur if intensity is misestimated when using the SCORAD; Surrogate markers of disease severity could overcome or supplement shortcoming of clinical scores in AD research [22]. As there are no other validated instruments for AD patients in TCM area, we believe that this instrument may be useful in daily clinical practice and scientific research, especially in the area of Complementary or Alternative Medicine (CAM).

The instrument has 15 items, with 12 rating items and 3 additional items. The 12 rating items fall within three domains: AD symptoms (n = 6 items), Heat (n = 4 items) and Mood (n = 2 items). Symptoms in the Heat and Mood domains are the main symptoms of a kind of *Zheng* (the *up-flaming of heart fire*) that is one of the most frequently occurring patterns in AD in Chinese medicine [23]. Three symptoms – itching, insomnia and dry skin – were duplicated in the ZRADSQ and SCORAD. However, the weight and scaling or calculation of the items in the two scales are different. In SCORAD [14], itching and insomnia are measured by a visual analogue scale (VAS) that ranges from 0 to 10 points for each item, whereas in the ZRADSQ, itching, insomnia, and dry skin are assessed on a four-point Likert-type scale. Further, although dry skin is evaluated with a four-point Likert-type scale in both SCORAD and the ZRADSQ, it is evaluated by the physician in SCORAD but is self-reported by patients in the ZRADSQ. The scaling and calculating methods of these two scales vary because of differences in the underlying theory that guided their development. The Pearson’s correlation coefficient between the ZRADSQ and SCORAD was 0.40, indicating a moderate correlation and implying that the ZRADSQ is only partially related to SCORAD. The observed correlations indicate good construct validity. The internal consistency and split-half consistency indicate that the ZRADSQ has good psychometric properties. However, the test-retest reliability is not so robust due to the short retest timeframe, and the responsiveness in the age equal or less than 7 years group is not good enough.

This study has several limitations. First, for practical reasons, the sample size for the validation of the questionnaire was fairly small. And more than half of the participants (57%) were less than 14 years old which may potentially limit the generalizability of the ZRADSQ. The results thus need to be further evaluated in a larger AD population, and should also be applied in an international context, including a control group of healthy participants, as further validation. Second, although the ZRADSQ is a simple and self-reported instrument, it may be difficult for patients under seven years old to understand and complete. We did not evaluate the consistency between the self-reported and parent-reported questionnaires. Third, we did not random the order of the instruments’ delivering, and thus could not avoid the possibility that the results may have been affected by the fixed order in instruments’ reporting. Fourth, we did not give any definitions of the terms used in the ZRADSQ. Instead, the participants were guided to choose the answer for each item that best represented their feelings or the symptoms that they had noticed. This means of obtaining responses may be more feasible for subjective items than for objective items. Items such as dark urine or constipation that can be described objectively may be biased when reported based on individual perceptions. Finally, we did not establish a mean score for the instrument in an AD population, which we leave for future research.

## Conclusions

We have developed and validated an instrument to assess *Zheng*-related symptoms in patients with AD. The instrument demonstrates promising reliability and validity. It is a simple questionnaire that is easy to understand and complete, and will thus be a suitable instrument for hospital or population based research on AD. Especially when the interventions are complementary therapies, it can be used to determine whether the efficacy or effectiveness of a certain therapy on AD is connected with the change of TCM symptoms, and whether *Zheng*-specific or symptom-specific treatments relieve the symptom that is most bothersome the patient. Future work will focus on instrument validation in a larger population and in an international context.

## Abbreviations

ZRADSQ: *Zheng*-related atopic dermatitis symptom questionnaire; TCM: Traditional Chinese medicine; AD: Atopic dermatitis; CAM: Complementary or alternative medicine; SCORAD: Severity scoring of atopic dermatitis; EASI: Eczema area and severity index; POEM: Patient-oriented eczema measure; CFA: Confirmatory factor analysis; RMSEA: Root mean square error of approximation; SES: Standardized effect size; SRM: Standardized response mean; CFI: Comparative fit index; VAS: Visual analogue scale.

## Competing interest

The authors declare that they have no competing interests.

## Authors’ contributions

DRW provided direct input into the design and execution of the study and the preparation and review of the manuscript. CJH provided direct oversight of the study conduct, conducted literature reviews, and contributed to the preparation and review of the manuscript. XMM, JFL coordinated and administered the study including pretesting, patient recruitment, data collection. JXC managed data entry, psychometric analysis, and statistical aspects of the study (with DRW), and contributed to the preparation and review of the manuscript. CL, HLZ, and HYL provided operational support and participated in the data collection processes, participated in instrument design, and reviewed the final manuscript. DCC provided oversight of the project and participated in the item reduction and pretesting processes, and instrument design, and contributed to the preparation and review of the final manuscript. All authors read and approved the final manuscript.

## Supplementary Material

Additional file 1 ***Zheng*-related atopic dermatitis symptom questionnaire (ZRADSQ).** Click here for file
