# Supplementary material for: Development and initial validation of a traditional Chinese medicine symptom-specific outcome measure: a Zheng-related atopic dermatitis symptom questionnaire (ZRADSQ)
Source: Health Qual Life Outcomes. 2013 Dec 21;11:212. doi: 10.1186/1477-7525-11-212 (PMC3881015; doi:10.1186/1477-7525-11-212)
Supplement: Additional file 1 — Zheng-related atopic dermatitis symptom questionnaire (ZRADSQ). [file 1477-7525-11-212-S1.doc]

**特应性皮炎主症量表**

***Zheng*-Related Atopic Dermatitis Symptom Questionnaire (ZRADSQ)**

| 姓 名: |  |  | 性 别: | □男 | □女 |
| --- | --- | --- | --- | --- | --- |
| 出生年月日: | 年 月 日 |  | 填写日期: | 年 月 日 | |

| **⑴瘙痒 ⑵又痒又痛 ⑶瘙痒夜间加重 ⑷皮肤干燥 ⑸身体灼热 ⑹烦躁**  **⑺易怒 ⑻失眠 ⑼口干 ⑽口渴 ⑾小便黄 ⑿便秘** |
| --- |

填写以下问题时，请先在方框中寻找适合的症状，如果方框内没有包含您的选项，则可以在横线上直接填写。

**1. 您（或您的孩子）患了特应性皮炎，现阶段最困扰您（或他/她）的症状（或最想解决的症状）是哪两个？**

**如果是复诊患者，请填写上次来诊时困扰您（或他/她）的症状现阶段是怎样的？（如果上次没有，则此项可以不填）**

**第一困扰的症状：**________________________

该症状的困扰程度（请在下列线段中选择最适合处打“/”）：

| 没有困扰├─────┴─────┼─────┴─────┤极大困扰  0 10 □ |
| --- |
|

**第二困扰的症状：**________________________，

该症状的困扰程度（请在下列线段中选择最适合处打“/”）：

| 没有困扰├─────┴─────┼─────┴─────┤极大困扰  0 10 □ |
| --- |
|

**2. 如果您是复诊病人，现阶段，有新的症状出现吗？ □没有=0，□有=1 （首诊不用填）**

**如果有，则该症状是：_______________ ，**（请先在方框中寻找合适答案，如均不符合，请按实际症状填写）

该症状的困扰程度（请在下列线段中选择最适合处打“/”）：

| 没有困扰├─────┴─────┼─────┴─────┤极大困扰  0 10 □ |
| --- |

3.请根据您的感受，在下面最接近的选项处打“√”。

| **条目/评分** | **没有** | **轻度** | **中度** | **重度** |
| --- | --- | --- | --- | --- |
| **⑴瘙痒** | □ 0分 | □ 1分 | □ 2分 | □ 3分 |
| **⑵又痒又痛** | □ 0分 | □ 1分 | □ 2分 | □ 3分 |
| **⑶瘙痒夜间加重** | □ 0分 | □ 1分 | □ 2分 | □ 3分 |
| **⑷皮肤干燥** | □ 0分 | □ 1分 | □ 2分 | □ 3分 |
| **⑸身体灼热** | □ 0分 | □ 1分 | □ 2分 | □ 3分 |
| **⑹烦躁** | □ 0分 | □ 1分 | □ 2分 | □ 3分 |
| **⑺易怒** | □ 0分 | □ 1分 | □ 2分 | □ 3分 |
| **⑻失眠** | □ 0分 | □ 1分 | □ 2分 | □ 3分 |
| **⑼口干** | □ 0分 | □ 1分 | □ 2分 | □ 3分 |
| **⑽口渴** | □ 0分 | □ 1分 | □ 2分 | □ 3分 |
| **⑾小便黄** | □ 0分 | □ 1分 | □ 2分 | □ 3分 |
| **⑿便秘** | □ 0分 | □ 1分 | □ 2分 | □ 3分 |

评分说明:

0=无任何症状；

1=轻度症状（有症状但影响不大）；

2=中度症状（症状有影响，但不妨碍日常活动）；

3=重度症状（症状有影响，并且妨碍到日常活动）。

***Zheng*-Related Atopic Dermatitis Symptom Questionnaire (ZRADSQ)**

| **Name:** |  |  | **Gender:** | **□Male** | **□Female** |
| --- | --- | --- | --- | --- | --- |
| **D O B:** | / / |  | **D a t e:** | / / | |

| **⑴Itching ⑵Itching accompanied by pain ⑶Itching aggravated at night ⑷Dry skin**  **⑸Burning skin ⑹Fidgeting ⑺Irascibility ⑻Insomnia**  **⑼Mouth dryness ⑽Thirst ⑾Dark urine ⑿Constipation** |
| --- |

(Please indicate your response on the following blanks by first identifying the symptoms in the above box and recording the numbers of the symptoms in the corresponding spaces if it suits your feelings, or you can write down the symptoms directly in the spaces below if there is no answer suits your feelings.)

**1. Please record the symptom that bothers you the most as *Primary bothersome Symptom* and another symptom, if there is any, which secondly bothers you as *Secondary bothersome Symptom* during the last week.**

**If this is not the first time you visit the doctor, please record the present situation of the symptoms you fulfilled last time in the corresponding blanks. *(If no symptom was recorded last time, you can skip to the next question.)***

**Primary (1^st^)** bothersome symptom:

Please mark a “/” through the line below that most accurately represents the level of bothersome of this symptom.

| No bothersome├─────┴─────┼─────┴─────┤Most bothersome  0 10 □ |
| --- |
|

**Secondary (2^nd^)** bothersome symptom:

Please mark a “/” through the line below that most accurately represents the level of bothersome of this symptom.

| Not bothersome├─────┴─────┼─────┴─────┤Most bothersome  0 10 □ |
| --- |

**2. If this is not the first time you visit the doctor, please record if there is any bothersome symptom newly occurs during the last week?**

□***No***=0，□***Yes***=1  *(New patients need* ***not*** *to fill.)*

If the answer is ***“Yes”***, then the new symptom is:_____________________

*(Please first find the symptoms in the box above, or you can write down the symptom directly if there is no answer suits your feelings.)*

And please mark a “/” through the line below that most accurately represents the level of bothersome of this symptom

| No bothersome├─────┴─────┼─────┴─────┤Most bothersome  0 10 □ |
| --- |

**3. What is the overall severity of these symptoms during the last week? (Please choose one number of each symptom with “√”** to indicate its severity)

| **Item/Score** | **None** | **Mild** | **Moderate** | **Severe** |
| --- | --- | --- | --- | --- |
| **⑴ Itching** | □ 0 | □ 1 | □ 2 | □ 3 |
| **⑵ Itching accompanied by pain** | □ 0 | □ 1 | □ 2 | □ 3 |
| **⑶ Itching aggravated at night** | □ 0 | □ 1 | □ 2 | □ 3 |
| **⑷ Dry skin** | □ 0 | □ 1 | □ 2 | □ 3 |
| **⑸ Burning skin** | □ 0 | □ 1 | □ 2 | □ 3 |
| **⑹ Fidgeting** | □ 0 | □ 1 | □ 2 | □ 3 |
| **⑺ Irascibility** | □ 0 | □ 1 | □ 2 | □ 3 |
| **⑻ Insomnia** | □ 0 | □ 1 | □ 2 | □ 3 |
| **⑼ Mouth dryness** | □ 0 | □ 1 | □ 2 | □ 3 |
| **⑽ Thirst** | □ 0 | □ 1 | □ 2 | □ 3 |
| **⑾ Dark urine** | □ 0 | □ 1 | □ 2 | □ 3 |
| **⑿ Constipation** | □ 0 | □ 1 | □ 2 | □ 3 |

**Key to symptoms:**

0= no symptoms,

1= mild symptoms (symptoms that are present but not particularly bothersome),

2= moderate symptoms (symptoms that are bothersome but do not interfere with daily activities),

3= severe symptoms (symptoms that are bothersome and interfere with daily activities).
